# Integrated profiling of iPSC-derived motor neurons carrying *C9orf72*, *FUS*, *TARDBP*, or *SOD1* mutations

**DOI:** 10.1016/j.stemcr.2025.102649

**Published:** 2025-10-02

**Authors:** Guo-ming Ma, Cong-cong Xia, Bo-yu Lyu, Jie Liu, Fang Luo, Ming-feng Guan, Jun-ying Wang, Li Sun, Lin Zhang, Yan Chen, Ying-wei Mao, Guo-qiang Yu, Wen-yuan Wang

**Affiliations:** 1 Interdisciplinary Research Center on Biology and Chemistry, Shanghai Institute of Organic Chemistry, Chinese Academy of Science, Shanghai 200032, China; 2 Bradley Department of Electrical and Computer Engineering, Virginia Polytechnic Institute and State University, Arlington, VA 22203, USA; 3 The International Peace Maternity and Child Health Hospital, School of Medicine, Shanghai Jiao Tong University, Shanghai 200030, China; 4 Research Center for Aging and Medicine, Huashan Hospital, Fudan University, 12 Wulumuqi Zhong Road, Jing’an District, Shanghai 200040, China; 5 Department of Neurology & National Clinical Research Center for Aging and Medicine, Huashan Hospital, Fudan University, 12 Wulumuqi Zhong Road, Shanghai 200040, China; 6 Penn State University, 214 Life Sciences Building, University Park, PA 16802, USA; 7 Department of Automation, Tsinghua University, Beijing 100084, China; 8 Department of Rehabilitation Medicine, Huashan Hospital, Fudan University, Shanghai 200040, China

**Keywords:** ALS, gene expression alterations, RNA sequencing, synaptic dysfunction

## Abstract

Here, we conducted temporal RNA sequencing (RNA-seq) profiling of human induced pluripotent stem cells (hiPSCs) and induced pluripotent stem cell (iPSC)-derived motor neurons (iMNs) carrying *C9orf72*, *FUS*, *TARDBP*, or *SOD1* mutations in both patients with amyotrophic lateral sclerosis (ALS) and healthy individuals. We discovered dysregulated gene expression and alternative splicing (AS) throughout iMN development and maturation, and iMNs with mutations in ALS-associated genes displayed enrichment of cytoskeletal defects and synaptic alterations from the premature stage to mature iMNs. Our findings indicate that synaptic gene dysfunction is a common molecular hallmark of familial ALS, which may result in neuronal susceptibility and progressive motor neuron degeneration. Analysis of upstream splicing factors revealed that differentially expressed RNA-binding proteins (RBPs) in iMNs from patients with ALS may cause abnormal AS events. Overall, our research provides a comprehensive and valuable resource for gaining insights into the shared mechanisms of familial ALS pathogenesis during motor neuron development and maturation in iMN models.

## Highlights

• A comprehensive map of gene expression and AS changes during ALS iMN differentiation
• Early synaptic dysfunction is a shared hallmark across various ALS gene mutations
• ALS iMNs show RBP dysregulation linked to abnormal alternative splicing events

## Teaser

In this article, Wen-yuan Wang and colleagues show that temporal transcriptome profiling of ALS iMNs reveals dynamic changes in gene expression and RNA splicing. Cytoskeletal defects and synaptic alterations emerge early and are shared characteristics across different ALS genes. Dysregulated RNA-binding proteins contribute to aberrant splicing, implicating splicing factor defects in ALS pathogenesis.

## Introduction

Amyotrophic lateral sclerosis (ALS) is a fatal neurodegenerative disorder that causes progressive weakness and muscle atrophy (Hardiman et al., 2017). More than 40 genes have been associated with ALS, with most cases linked to *C9orf72*, *SOD1*, *TARDBP*, or *FUS* mutations (Goutman et al., 2022). Recent studies have demonstrated that similar critical cellular pathways exhibit abnormalities in patients with ALS with various genetic backgrounds (Goutman et al., 2022). The use of induced pluripotent stem cell (iPSC)-derived motor neuron (iMN) models has dramatically expanded our ability to model ALS based on its clinical and genetic diversity (Ziff et al., 2023; Workman et al., 2023). The increased scope of ALS iMN research provides an opportunity to identify common motor neuron abnormalities across different ALS genetic backgrounds (Baxi et al., 2022).

Given the diversity and complexity of ALS pathogenesis, a crucial question arises: what molecular mechanisms, common or specific, are involved in ALS with different causative genes? The onset of ALS commonly occurs in mid-adulthood; however, the effects of ALS genes may manifest earlier in life (Taylor et al., 2016). Hence, we elucidated changes at the molecular level throughout motor neuron development and systematically examined the effects of ALS-causing genes on transcriptomes during motor neuron differentiation.

In this study, we generated fibroblast cell lines from patients with ALS harboring *C9orf72*, *FUS*, *TARDBP*, and *SOD1* mutations; reprogrammed the cells into iPSCs; and then differentiated them into motor neurons. We addressed common and specific gene expression changes, alternative splicing (AS) dysregulation, and specific splicing factors regulating AS events throughout motor neuron development and progression in patients with *C9orf72*-ALS, *FUS*-ALS, *TARDBP*-ALS, or *SOD1*-ALS using temporal RNA sequencing (RNA-seq) profiling.

## Results

### Generation of iPSCs and differentiation of functional iMNs from patients with ALS

**Figure 1:**
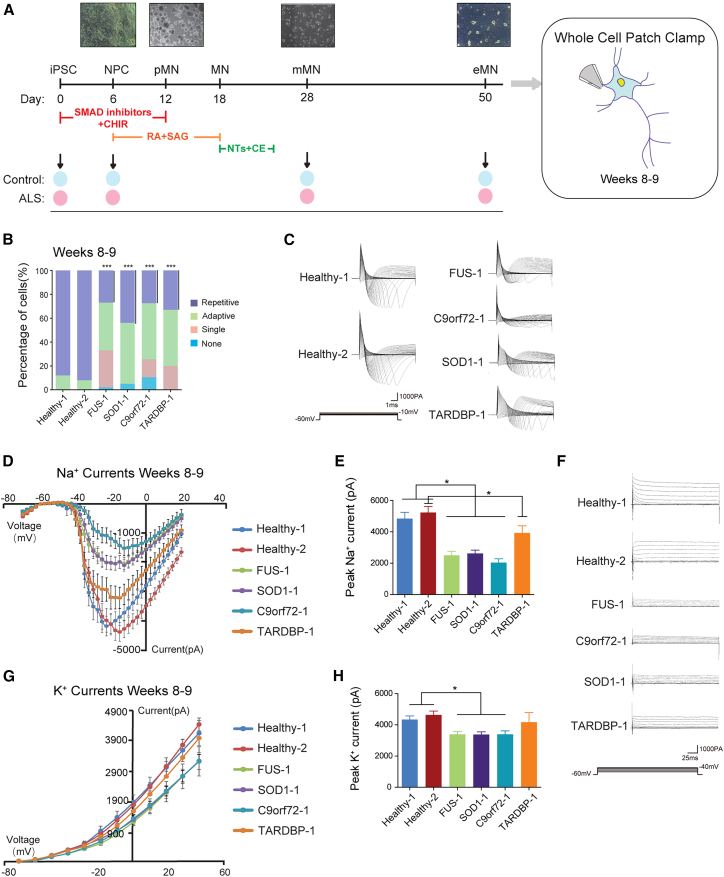
Generation of iPSCs and differentiation of functional iMNs from patients with ALS (A) Schematic of the differentiation process. (B) Proportion of cells in each AP firing category in iMNs from Healthy-1, Healthy-2, *FUS*-1, *SOD1*-1, *C9orf72*-1, or *TARDBP*-1 lines at weeks 8–9 post plating (Student’s t test; ^∗∗∗^*p* < 0.001). (C) Raw data of fast, inactivating Na^+^ currents. (D) The current-voltage relationships of peak Na^+^ currents. (E) Barplot of peak fast, inactivating Na^+^ currents (Student’s t test; ^∗^*p* < 0.05; mean ± SEM). (F) Raw data of persistent K^+^ currents from iMNs. (G) The current-voltage relationships of peak K^+^ currents. (H) Barplot of peak K^+^ currents (Student’s t test; ^∗^*p* < 0.05; mean ± SEM). For (B)–(H), *n* represents the number of individual cells recorded per line (cells from each cell line were derived from three independent batches of differentiation experiments): Healthy-1 (*n* = 58), Healthy-2 (*n* = 61), *FUS*-1 (*n* = 52), *SOD1*-1 (*n* = 41), *C9orf72*-1 (*n* = 47), or *TARDBP*-1 (*n* = 41).

We generated iPSCs from nine fibroblasts of patients with ALS carrying *SOD1* (*SOD1*-1, *SOD1*-2, *SOD1*-3, and *SOD1*-4), *TARDBP* (*TARDBP*-1 and *TARDBP*-2), *FUS* (*FUS*-1 and *FUS*-2), or *C9orf72* gene mutations, and four fibroblasts from healthy individuals (Figures S1A–S1C; Table S1). We obtained differentiated spinal iMNs (Figure 1A), which resulted in a highly near-pure population of PAX6^+^ and NESTIN^+^ neural progenitor cells (NPCs) (Figure S2A) in 6 days, OLIG2^+^ MN progenitors (>98%, Figure S2B) in 12 days, ISL1^+^ and HB9^+^ iMNs (>80%, Figures S2C and S2D) in 23 days, and CHAT^+^-maturing iMNs in 28 days (Figure S2E). The efficiency of iMN differentiation was similar between the control and ALS subgroups. We observed the formation of neuromuscular junctions (Figure S2F). Healthy iMNs were electrophysiologically active, as evidenced by their ability to elicit short-lasting action potentials in response to depolarizing current injection in current-clamp recording (Figure S2G), suggesting that they were fully functional iMNs. Taken together, these results indicate the successful development of an iPSC-based human spinal motor neuron disease model of ALS.

### Functional perturbation of iMNs derived from patients with ALS

We then investigated whether these ALS iMNs exhibit functional perturbations (Figure 1A). The number of cells able to fire action potentials (APs) was significantly decreased in ALS iMNs at weeks 8–9 (Figures 1B and S2H), especially the cells that could fire APs repetitively, indicating a reduction in the ability of iMNs to sustain a high rate of electrical activity over time and the impairment of their physiological functions.

We conducted tests on voltage-activated currents during AP generation in order to better understand the reason for the decrease in the output of AP in ALS iMNs. Our investigation began with fast, inactivating Na^+^ currents, which are responsible for the upstroke of the AP (Figure 1C). Our results revealed a gradual decline in Na^+^ currents in ALS iMNs (Figures 1D and S2I). Peak Na^+^ currents were significantly decreased in *FUS*, *SOD1*, and *C9orf72* iMNs compared to control groups. A slightly decreasing trend was observed in *TARDBP* iMNs (Figure 1E).

Then, we investigated whether these results indicated a more general reduction in voltage-activated currents in ALS iMNs. We measured persistent K^+^ currents (Figure 1F). Our findings revealed a progressive loss in peak K^+^ currents in ALS iMNs (Figures 1G and S2J). iMNs harboring *SOD1*, *FUS*, or *C9orf72* mutations exhibited significantly lower K^+^ currents than controls. iMNs derived from patients with *TARDBP-ALS* showed a slight reduction in K^+^ currents (Figure 1H). Our data demonstrate progressive loss of both fast, inactivating Na^+^ currents and persistent, voltage-activated K^+^ currents in iMNs from patients with ALS. It is plausible that the loss of AP output and the reduction in voltage-activated currents underlie the progressive functional decline in ALS iMNs.

### Transcriptomic disturbances in iMN development under the genetic background of ALS

**Figure 2:**
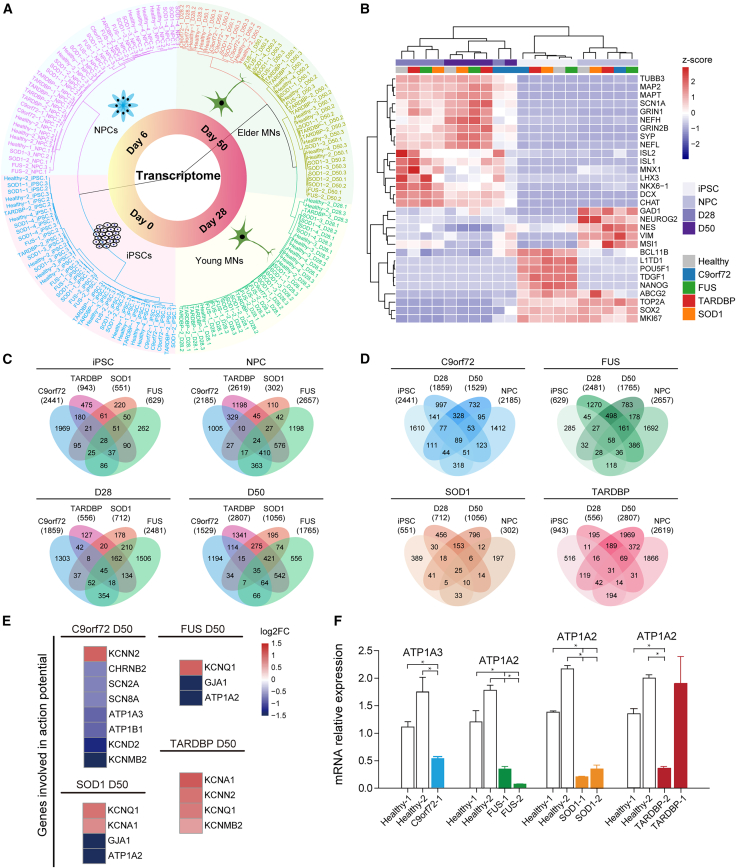
Transcriptomic disturbances during iPSC-derived motor neurogenesis on an ALS genetic background (A) Hierarchical clustering of all samples by transcriptomes. (B) The normalized average expression of selected marker genes. (C) Overlap of differentially expressed genes (DEGs) of ALS subgroups compared to healthy controls (FDR < 0.05, |fold change| ≥ 1.5). (D) Overlap of DEGs across four iMN developmental stages in each ALS subgroup (FDR < 0.05, |fold change| ≥ 1.5). (E) Heatmap of genes related to AP generation showing expression changes in mature iMNs. (F) The expression changes of genes related to AP generation on day 50 measured by qPCR (*n* = 3 independent experimental batches, Student’s t test; ^∗^*p* < 0.05, ^∗∗^*p* < 0.01, ^∗∗∗^*p* < 0.001, ^∗∗∗∗^*p* < 0.0001; mean ± SEM).

We performed RNA-seq from iPSCs, NPCs, day 28 iMNs, and day 50 iMNs in order to investigate ALS-related transcriptomic changes during iMN development and maturation. Hierarchical clustering revealed that gene expression changes were primarily regulated by developmental stage within the motor neuron lineage, rather than by genetic background (Figure 2A). Specific marker genes were highly expressed in different stages (Figure 2B).

We then investigated how ALS genes commonly and differentially affect changes in gene expression in iMNs. A significant number of genes exhibited altered expression levels in various ALS subgroups (Figure 2C). Further, many genes exhibited altered expression patterns throughout the early developmental stages of iMNs, which persisted into later stages, the progressive disease state (Figure 2D). Therefore, it is important to elucidate the molecular mechanisms that are commonly and temporally affected by *SOD1*, *FUS*, *TARDBP*, or *C9orf72* mutations to gain a better understanding of ALS pathogenesis.

We analyzed gene expression changes on day 50 to determine the root cause of the abnormal electrophysiological properties observed earlier. The results revealed significant expression changes of many genes involved in generating APs in ALS iMNs (Figure 2D), particularly subunits of voltage-gated sodium channels (e.g., *SCN2A*), subunits of voltage-gated potassium channels (e.g., *KCNQ1* and *KCNA1*), and catalytic subunits of Na^+^/K^+^-ATPase (e.g., *ATP1A3* and *ATP1A2*). These genes play crucial roles in establishing and maintaining the electrochemical gradients of Na^+^ and K^+^ across plasma membranes. Notably, *ATP1A2* was downregulated in both *FUS* and *SOD1* iMNs, and *ATP1A3* was downregulated in *C9orf72* iMNs (Figure 2E). The dysregulation of these genes was further validated by quantitative PCR (Figure 2F; Table S2). Intriguingly, *ATP1A2* was also downregulated in *TARDBP*-2 lines but not in *TARDBP*-1 lines, in line with electrophysiological data showing no significant differences between iMNs from *TARDBP*-2 lines and healthy lines. These results strongly suggest that the downregulation of Na^+^/K^+^-ATPases is a common molecular mechanism underlying ALS-associated neuronal dysfunction caused by ALS genes, thereby directly linking our findings to the disease state.

### ALS iMNs exhibit dysregulated neuronal function across genetic backgrounds

**Figure 3:**
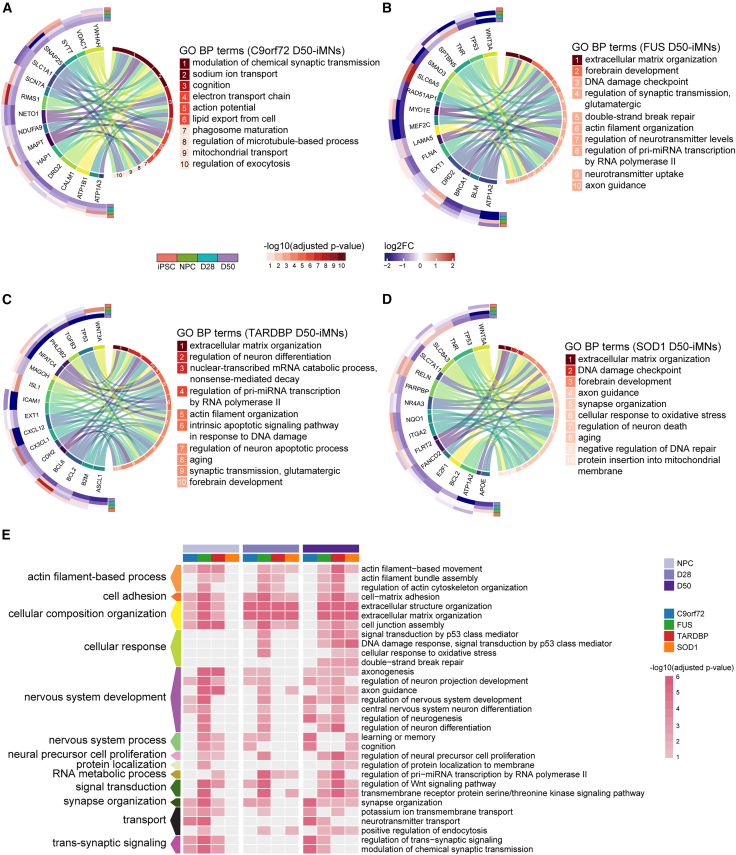
Distinct and common transcriptome alterations in mature iMNs across four ALS subgroups (A–D) Significantly dysregulated genes and Gene Ontology (GO) enrichment in ALS-*C9orf72* (A), ALS-*FUS* (B), ALS-*TARDBP* (C), and ALS-*SOD1* (D) iMNs on day 50, respectively (adjusted *p* value < 0.05). (E) The enrichment of selected GO terms (adjusted *p* value < 0.05).

We compared gene expression levels in day 50 iMNs to investigate possible connections among different ALS genes involved in ALS pathogenesis. We identified unique differentially expressed genes (DEGs) implicated in each ALS subgroup. Using Gene Ontology (GO) enrichment analysis, we identified 10 significantly enriched biological processes (BPs), ALS-*C9orf72*, ALS-*FUS*, ALS-*TARDBP*, and ALS-*SOD1*, as presented in Figure 3. We found that ALS-*C9orf72* affected synaptic transmission and ion transport (Figure 3A). iMNs with ALS-*FUS* showed changes in extracellular matrix organization, synaptic transmission, and DNA damage (Figure 3B). ALS-*TARDBP* affected extracellular matrix organization, neuronal differentiation regulation, and nonsense-mediated decay (Figure 3C). iMNs with ALS-*SOD1* showed changes in extracellular matrix organization, DNA damage, and oxidative stress response (Figure 3D). Our comparisons of significant BPs among the four ALS genes identified several common dysregulated cellular functions. From the NPCs to the day 50 iMNs, the cytoskeleton, cell adhesion, cellular composition organization, synaptic function, cellular response, and neuronal development were enriched in two or more ALS subgroups, indicating that common dysregulated neuronal mechanisms may be involved among these ALS-causative genes (Figure 3E). These results suggest that transcriptome alterations are usually distinct in iMNs derived from patients with ALS with mutations in *C9orf72*, *FUS*, *TARDBP*, or *SOD1* but may overlap to some extent in mature iMNs with perturbed electrophysiological properties.

### ALS genes result in dysregulated neuronal function in premature iMNs

**Figure 4:**
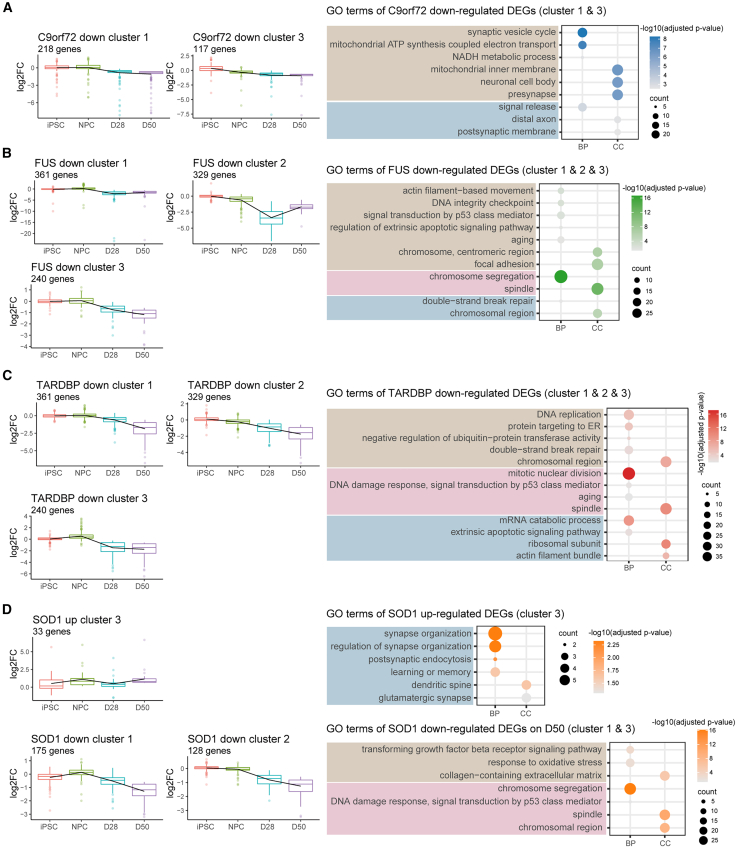
Temporal transcriptome alterations in ALS subgroups during iMN development and maturation Left, trends of log2 fold change values of DEGs from the selected clusters in ALS-*C9orf72* (A), ALS-*FUS* (B), ALS-*TARDBP* (C), and ALS-*SOD1* (D). Right, enriched GO terms in corresponding clusters (adjusted *p* value < 0.05).

We then examined time-dependent gene expression changes in ALS subgroups. We clustered the log2-fold change values of DEGs across all stages of development and performed GO analysis of DEGs from each cluster. Results showed that in ALS-*C9orf72*, cluster 1 displayed a downregulated pattern from day 28. The genes in this cluster were significantly enriched in the synaptic area mitochondrial function (Figure 4A). The DEGs in cluster 3 were mainly downregulated from the NPC stage, with significant localization in the distal axon and pre-synapse (Figure 4A). These findings suggest that *C9orf72* mutations cause abnormalities in the synapse and mitochondria from early stages, persisting into mature iMNs. In *TARDBP* iMNs, DEGs in clusters 1, 2, and 3 were significantly downregulated on day 50, and these genes already showed a trend of downregulated expression from day 28 (Figure 4B). These DEGs were enriched in RNA metabolism, protein localization, and DNA damage response. In *FUS* iMNs, clusters 1, 2, and 3 showed a downregulation trend from day 28. *FUS* mutations lead to functional disorders such as p53 signal transduction and cytoskeleton (Figure 4C). These results suggest that *TARDBP* and *FUS* mutations can cause dysregulation of p53 signal transduction in the early stages of iMN maturation. In *SOD1* iMNs, clusters 1 and 2 represented downregulated DEGs from day 28; they were enriched in the transforming growth factor β receptor signaling pathway and DNA damage response (Figure 4D). In contrast, DEGs in cluster 3 showed an upregulation trend from the NPC stage. Moreover, they are enriched in synapse organization, postsynaptic endocytosis, learning, and memory (Figure 4D). These results suggest that *SOD1* mutations cause abnormal synaptic function in the early stages. Overall, our findings revealed that ALS genes can affect many essential iMN functions from the early stages, which may provide potential early targets for clinical intervention for ALS disease.

### Transcriptome alterations in ALS iMNs reveal the intricate dynamics of signaling pathways

We analyzed gene expression changes during iMN development and identified DEGs between two consecutive developmental stages to understand the temporal dynamics of the process. Interestingly, we found that *FUS*, *TARDBP*, and *SOD1* mutations had more consistent effects on gene expression levels (Figures S3A–S3C). We also performed a signaling Pathway RespOnsive GENes (PROGENy) analysis to investigate the activated pathways during iMN development and maturation. Our findings revealed that the WNT pathway activity was increased in healthy and ALS subgroups. However, the JAK-STAT pathway showed decreased activity from iPSCs to NPCs (Figure S3D), and the epidermal growth factor receptor and mitogen-activated protein kinase pathways had decreased activity across all of the subgroups during the transition from NPCs to mature iMNs (Figure S3E).

At the mature iMN stage, our research revealed significant differences in the activities of signaling pathways among healthy and ALS subgroups. Specifically, we observed that JAK-STAT pathway activation was present only in healthy iMNs, whereas inflammation-related nuclear factor κB (NF-κB) and tumor necrosis factor alpha (TNF-α) pathways were significantly activated in *FUS* and *SOD1* iMNs (Figures S3F–S3J). These findings underscore the substantial divergent expression changes among ALS subgroups when transitioning from day 28 iMNs to day 50 iMNs, highlighting the divergent pathway activities between healthy and ALS iMNs.

### Healthy and ALS-specific AS events affect neuronal function in ALS iMNs during differentiation

**Figure 5:**
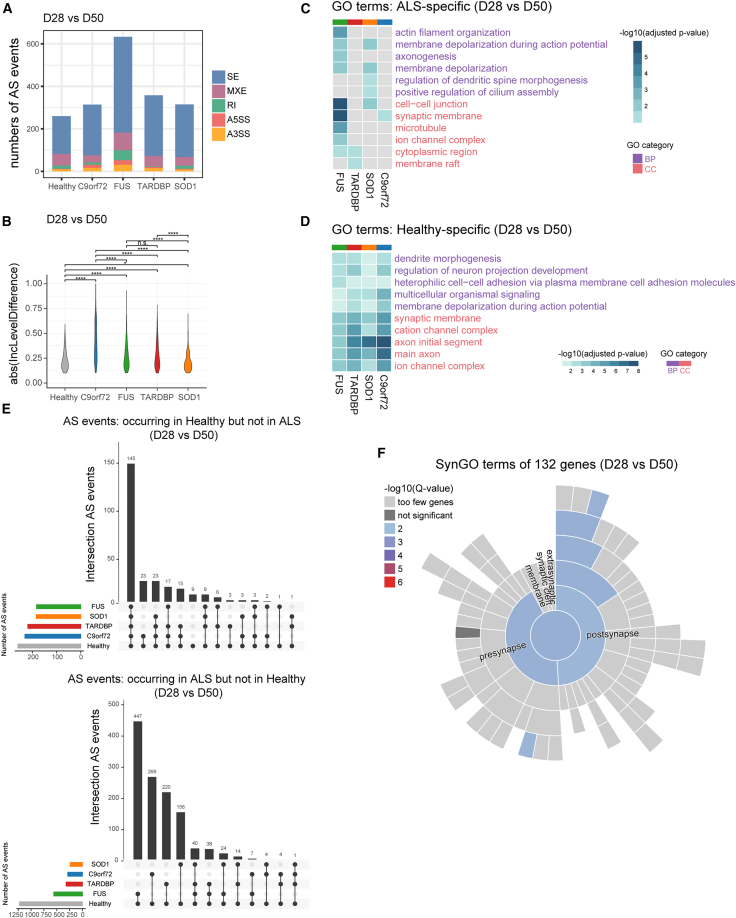
Aberrant splicing events in day 50 iMNs compared to day 28 iMNs (A) Number of splicing types. (B) Distribution of the absolute inclusion level differences in significant alternative splicing (AS) events (Wilcoxon signed-rank test; ^∗^*p* < 0.05, ^∗∗^*p* < 0.01, ^∗∗∗^*p* < 0.001, ^∗∗∗∗^*p* < 0.0001, n.s., not significant). (C) The common GO terms associated with genes were only differentially alternatively spliced in ALS iMNs. (D) Common GO terms associated with genes were only differentially alternatively spliced in healthy iMNs. (E) Overlaps of splicing events were significantly altered in healthy controls but not in ALS subgroups (left) and vice versa (right). (F) The sunburst plot displays SynGO annotations for genes with AS in healthy controls but does not show the same splicing events in ALS subgroups.

In patients with ALS, abnormal AS changes occur in the primary motor cortex, which may lead to protein dysfunction and worsen the progression of the disease (Martinez et al., 2016; Lin et al., 2016). We conducted AS analyses at different stages to investigate how AS was affected in ALS iMNs during their development and maturation (Figure 5A). We identified significant AS events, including skipped exons (SEs), mutually exclusive exons (MXEs), alternative 5′/3′ splice sites (A5SSs, A3SSs), and retained introns (RIs). GO analysis revealed that ALS- and healthy-specific alternatively spliced genes were enriched in regulating GTPase activity, dendritic or neuron projection, microtubules, and cell polarity from NPC to day 28 (Figure S4B).

Because ALS typically manifests functional deficits in motor neurons during the later stages of disease progression, we focused on AS events from day 28 iMNs to day 50 iMNs and identified 633, 358, 315, 314, and 260 significant AS events in the *FUS*, *TARDBP*, *SOD1*, *C9orf72*, and healthy subgroups, respectively (Figures 5A and S4A). Both the ALS and healthy subgroups exhibited an increase in the frequency of SE events during iMN terminal maturation (Figure S4A). In addition, compared to healthy controls, *FUS*, *TARDBP*, and *C9orf72* iMNs displayed significantly higher absolute inclusion levels of AS events (Figure 5B).

We analyzed the inclusion events that were significantly changed in the ALS and healthy subgroups (Figure S4C). We identified 556, 317, 239, and 283 AS events that were significant in *FUS*, *TARDBP*, *SOD1*, and *C9orf72* iMNs, respectively, while none were significant in the healthy subgroups. In contrast, we found 183, 219, 184, and 231 AS events to be healthy specific, respectively. Mutant-specific AS events were enriched in GO categories, such as actin filament organization and axonogenesis in *FUS* iMNs, regulation of dendritic spine morphogenesis in *SOD1* iMNs, and membrane depolarization in *FUS* and *SOD1* iMNs (Figure 5C). We also found that genes that underwent AS were present in various cellular components (CCs), including the synaptic membrane in *FUS* and *C9orf72* iMNs (Figure 5C).

Genes that display AS events specific to healthy subgroups exhibit more significant functional enrichment. For example, we observed BPs related to the regulation of neuron projection development, membrane depolarization during APs, and CCs related to the synaptic membrane, axon initial segment, and ion channel complex (Figure 5D). We also focused on genes with differential splicing between healthy and ALS subgroups and found 145 AS events occurring in healthy subgroups but not in ALS subgroups from days 28 to 50 (Figure 5E). Interestingly, iMNs with *FUS*, *TARDBP*, or *SOD1* mutations showed similar differences in exon inclusion levels on day 50 (Figures S4D and S4E).

Because aberrant AS events were synaptically enriched in each ALS subgroup, we used SynGO to investigate 132 genes exhibiting different splicing situations of 145 events (Figure 5E). Synaptic ontology terms demonstrated enrichment in these genes, including *NRXN2*, *SCN2A*, *LRRC7*, and others (Figure 5F). In conclusion, our study indicates that mutations in ALS-causative genes can lead to aberrant AS, particularly affecting genes specifically spliced in healthy subgroups, which, in turn, may impact synaptic and other neuronal functions during motor neuron maturation from days 28 to 50.

### Aberrant AS events occur in mature ALS iMNs

**Figure 6:**
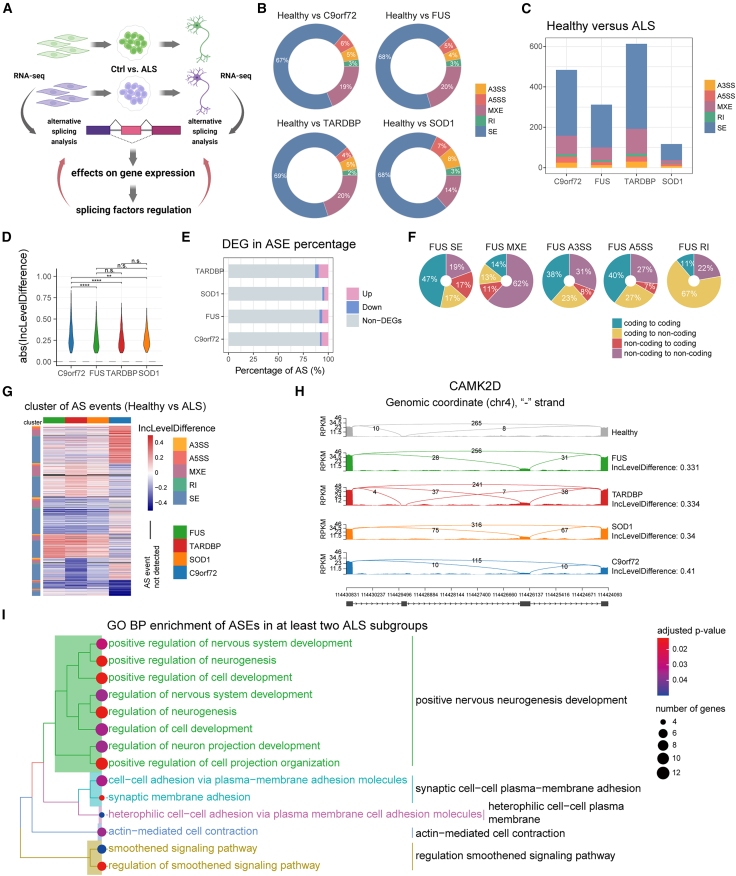
Alternative splicing alterations in day 50 iMNs from patients with ALS (A) Schematic illustrating the analysis workflow of alternative splicing. (B) Proportions of splicing types. (C) Number of splicing types. (D) Distribution of absolute inclusion-level differences (Wilcoxon signed-rank test; ^∗^*p* < 0.05, ^∗∗^*p* < 0.01, ^∗∗∗^*p* < 0.001, ^∗∗∗∗^*p* < 0.0001, n.s., not significant). (E) The ratio of up- and down-regulated genes and non-DEGs. (F) The distribution of annotated transcripts based on changes in their potential protein-coding ability due to AS events. (G) The inclusion-level differences in AS events significant in at least one of the four ALS subgroups from included (red) to excluded (blue). (H) The sashimi plot of *CADM1* and *CAMK2D* shows significant AS events in ALS subgroups. (I) Enriched BP terms of genes showing AS significant changes in at least two ALS subgroups.

We analyzed AS events between ALS and healthy subgroups to better understand the impact of *FUS*, *TARDBP*, *SOD1*, and *C9orf72* mutations on RNA splicing during disease progression (Figure 6A). We noticed significant changes in AS from NPCs to day 50 iMNs, with SE events accounting for approximately 60% of the AS events, which is consistent with prior studies (Figures 6B and S5A) (Luisier et al., 2018).

We observed the most significant changes in AS events in iMNs carrying *TARDBP* mutations, followed by *C9orf72* and *FUS* mutations on day 50 (Figures 6C, 6D, and S5D). The distribution of AS changes (exon inclusion levels) was similar among all of the ALS subgroups (Figures S5A and S5B). On day 50, approximately 10% of the genes with AS changes were DEGs in the ALS subgroups, with the proportion of upregulated genes being higher than that of downregulated genes (Figure 6E).

We investigated the coding potential of transcribed sequences to better understand the impact of AS on protein function. We mapped transcripts to splicing events to explore possible protein features affected by splicing. Results showed that changes in protein-coding ability were mainly preserved during SE and MXE events (Figures 6F and S5C). However, RI events caused significant changes in protein coding, converting them from noncoding to coding or vice versa (Figures 6F and S5C).

Next, we analyzed shared AS changes among different ALS subgroups. To determine significant differences in the inclusion level of AS events, we calculated Pearson’s correlation coefficients and found a strong correlation among ALS-*FUS*, ALS-*TARDBP*, and ALS-*SOD1* (Figure S5E). We grouped significantly differentially spliced events into seven distinct clusters and identified 1,308 AS events in at least one ALS subgroup on day 50 (Figure 6G). Of these, three significantly changed in four ALS subgroups, 28 in three, and 153 in two ALS subgroups.

Our study identified *CAMK2D* and *CADM1* as significant players in all of the ALS subgroups (Figures 6H, S6A, and S6B). CAMK2D plays a crucial role in the plasticity at glutamatergic synapses (Kool et al., 2019). Cell adhesion molecule 1 (CADM1) is a crucial facilitator of cell adhesion that is implicated in the genetic architecture of Attention-Deficit/Hyperactivity Disorder (ADHD) (Jin et al., 2019; De Araújo Lima et al., 2016).

We then focused on identifying changes in AS events in at least two ALS subgroups. Results showed that genes with AS changes were mainly localized in the presynaptic area and axons (Figure S5F), as well as enriched in the regulation of nervous system development (Figure 6I). These findings suggest that mutations in ALS-causative genes affect the splicing of genes related to essential neuronal functions, including synapse regulation, in day 50 iMNs.

### Splicing factors dysregulate AS transcription in mature iMNs

Recent studies have shown that most splicing occurs during transcription, and transcription factors (TFs) may influence splicing outcomes (Ullah et al., 2023). To investigate the role of TFs in splicing regulation in ALS iMNs on day 50, we identified TFs with aberrant splicing events. KMT2A was found to regulate AS events in *FUS*, *TARDBP*, and *SOD1* iMNs (Figures S7A and S7B), and recent evidence suggested that MeHg accelerated necroptotic cell death in SOD1-G93A cells via the Sp1/KMT2A complex (Guida et al., 2021).

RNA-binding proteins (RBPs) also play an important role in regulating gene expression. Dysfunctions in RBPs are associated with neurodegeneration (Lukong et al., 2008). Recent studies have highlighted the involvement of RBPs in ALS pathogenesis (Pham et al., 2020).

**Figure 7:**
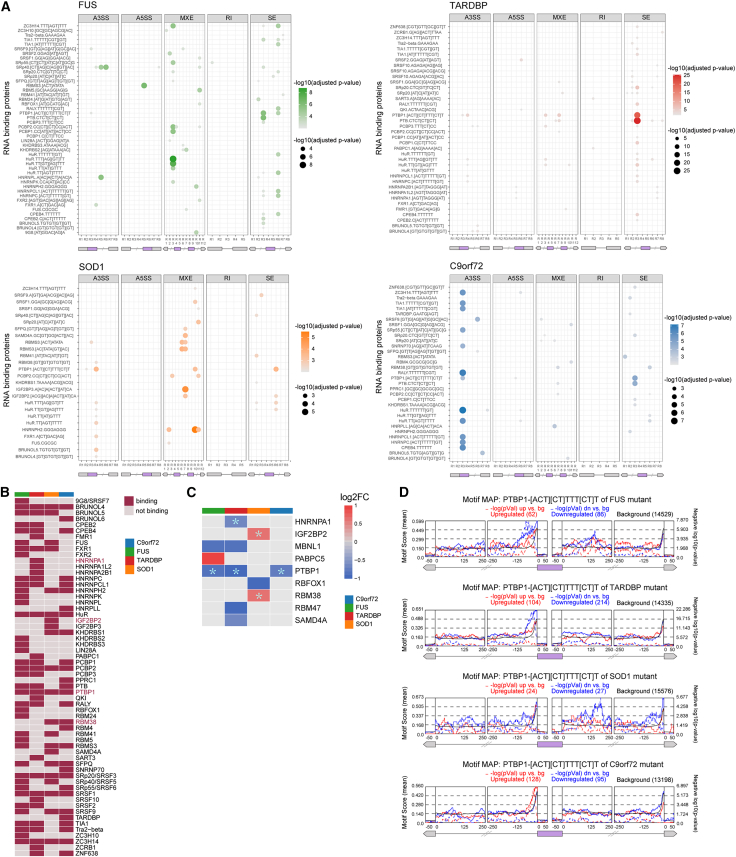
Splicing factor regulation of aberrant AS events in ALS iMNs on day 50 (A) Bubble plot of significantly enriched RNA-binding protein (RBP) binding motifs around AS events (adjusted *p* value < 0.001). The region (R) of each splicing event is numbered from R1 to R (*n*), 5′–3′ (bottom). Exons: purple boxes; up- and downstream exons: gray boxes. (B) The heatmap for motif binding overlaps of RBPs. (C) The log2 fold change value of significant differentially expressed RBPs. RBPs marked by “^∗^” are DEGs with binding sites in regions flanking alternative splicing events. (D) Positional distribution of the PTBP1-binding motif. The solid lines show the mean motif score, representing the percentage of nucleotides covered by the PTBP1-binding motif.

We conducted a binding motif enrichment analysis of 91 known RBPs to understand the molecular mechanisms underlying abnormal AS in ALS subgroups mediated by RBPs. Motif analysis showed that 60 RBPs bound with accompanying AS events in at least one ALS subgroup on day 50 (Figure 7B).

We observed that RNA-binding motifs (RBMs) are mainly enriched in various AS events specific to ALS subgroups (Figure 7A). Out of the 60 identified RBPs, five were reported as genes or genetic variants associated with ALS, according to the Amyotrophic Lateral Sclerosis online Database (ALSoD) criteria (Abel et al., 2012). These include FUS, HNRNPA1, HNRNPA2BA, TARDBP, and TIA1 (Figure 7B).

PTBP1, a splice factor belonging to the ubiquitously expressed heterogeneous nuclear ribonucleoprotein (hnRNP) subfamily (Bampton et al., 2020), exhibited approximately 1-fold downregulation in gene expression in ALS-*FUS*, ALS-*TARDBP*, and ALS-*C9orf72*. The binding motif of PTBP1 was enriched in regions flanking significant AS events across all of the ALS subgroups (Figures 7C and 7D). RBM analysis revealed that SE events in iMNs were typically enriched for PTBP1-binding sites within 100 nucleotides upstream of the skipped exon or 200 nucleotides downstream of the skipped exon (Figures 7A and 7D). Our results and recent findings highlight the potential role of PTBP1 downregulation in neurogenesis and neurodegenerative disease (Xue et al., 2016), which necessitates further research.

Besides, most of these RBPs reported in prior studies (Van Nostrand et al., 2020) exhibited significant downregulation, suggesting dysfunction in RBPs and their potential contribution to dysregulated AS in ALS iMNs. Our findings suggest that differentially expressed RBPs or other upstream transcription regulators in ALS iMNs affect abnormal AS events.

## Discussion

In this study, we systematically characterized transcriptomic changes in ALS iMNs with *C9orf72*, *FUS*, *TARDBP*, or *SOD1* mutations at four temporal time points (Figure 1). These stages were carefully selected based on prior literature and validated within our system. iPSCs serve as a baseline for identifying early lineage-specific changes in gene expression. The NPC stage enabled us to detect early transcriptomic disruptions associated with ALS mutations before apparent neuronal differentiation occurred. At day 28, iMNs exhibited key markers and developed axonal and dendritic morphology, indicating functional neuronal identity. The extension of differentiation to day 50 resulted in the acquisition of more mature features, including the formation of neuromuscular junction-like structures and the ability to generate fire action potentials. This stage is particularly significant for studying long-term ALS-associated phenotypes, including age-related degeneration.

Our findings provide insights into the cellular pathways underlying the progression of ALS and the pathomechanisms by which motor neurons progressively degenerate. We elucidated the representative BPs in mature iMNs that are disrupted by each ALS gene (Figure 3). These findings not only indicate the specific pathological mechanisms involved in different ALS genes but also suggest that the treatment of ALS should be tailored to the genetic cause of patients with ALS.

We discovered several BP categories that were significantly enriched in more than three ALS subgroups in mature iMNs (Figure 3E). These data indicate that dysregulation of the cytoskeleton, cell adhesion, cellular composition organization, synaptic function, cellular response, and neuronal development may begin early in ALS and persist in later stages of the disease, playing essential roles in the complex pathology networks (Figure 3E).

Our analysis also revealed the activation of inflammation-related NF-κB and TNF-α pathways in ALS iMNs on day 50 (ALS-*FUS* and ALS-*SOD1*). Notably, activation was less pronounced in ALS-*SOD1* than in ALS-*FUS* (Figure S3F). The potential role of neuroinflammation has gained significant attention in ALS research. Our findings suggest that ALS genes promote neuroinflammation at the transcriptomic level in mature iMNs.

Prior studies have reported dynamic changes in AS in mammalian brains and human iPSC-based models during developmental stages or in mature motor neurons (Ziff et al., 2023; Luisier et al., 2018). However, our understanding of the regulatory mechanisms of AS across motor neuron differentiation shared by different mutants is currently limited. We investigated AS events and examined the different regulatory mechanisms during iMN development and maturation. From days 28 to 50 of iMN maturation, genes with abnormal AS events were functionally overrepresented in neuron projection development and membrane depolarization during AP. These genes were also significantly present in CCs related to the synaptic membrane, axon initial segment, and ion channel complex, which are central to ALS motor neuron pathophysiology (Figure 5D). Further, genes exhibiting AS only in healthy subgroups were associated with pre- or post-synapse identified in SynGO (Figures 5E and 5F). One specific example is the neurexins (NRXN1, NRXN2, and NRXN3), a family of proteins that serve as cell adhesion molecules and receptors in the vertebrate nervous system. Mutations in the neurexin family have been identified in patients with autism spectrum disorder and schizophrenia (Tromp et al., 2021).

Specifically, we found that ALS genes induce more aberrant AS events in mature iMNs, which could have profound implications for our understanding of the disease. Further, it is worth noting that most of these events did not necessarily affect gene expression at a significant level (Figures 6E and 6F), suggesting that assessing only gene expression without considering actual isoform usage can provide limited information about the transcriptomic changes that affect disease biology.

Our analysis identified MXE events in *CAMK2D* on day 50 (Figures 6H and S6B). The *CAMK2D* gene produces the delta chain of the CAMK2 enzyme. CAMK2 is critical for calcium signaling, synaptic plasticity, and memory formation (Kool et al., 2019). A significant alteration in the splicing patterns of *CAMK2D* in all ALS iMNs may represent an innovative target for further studies.

Recent discoveries using *in vivo* and *in vitro* models of ALS have revealed that early synaptic dysfunction occurs before motor neuron degeneration symptom manifestation. This observation is further supported by postmortem analysis of tissues from a patient with ALS (Fischer et al., 2004). Further studies have shown that patients with ALS in the early stages of the disease exhibit signs of corticospinal degeneration, loss of lower motor neurons, and altered excitability of surviving motor units, whereas neuromuscular junctions remain functional (Devlin et al., 2015; Gelon et al., 2022; Marchand-Pauvert et al., 2019; Wainger et al., 2014; Martínez-Silva et al., 2018). Furthermore, analysis of a comprehensive collection of 429 iMNs across 15 datasets revealed that dysregulated synaptic signaling pathways were linked to both aberrantly expressed genes and AS events (Ziff et al., 2023; Workman et al., 2023).

Consistent with these studies, we also observed that disease-associated gene expression changes are not limited to mature iMNs but begin as early as the NPC stage. For example, GO analysis of DEGs revealed dysregulation of BPs related to cytoskeleton and synaptic organization in ALS subgroups from the NPC stage (Figures 3E and 4). In parallel, genes exhibiting aberrant AS changes were enriched in pathways regulating cytoskeletal functions from NPC to day 28 (Figure S4B). We noticed that genes with AS changes involved in at least two ALS subgroups on day 50 demonstrate significant enrichment in synaptic dysfunction (Figures 6I and S5F). Further, the temporal splicing alterations caused by ALS genes align with the electrophysiological properties illustrated in Figure 2, and the impacted molecular pathways during iMN maturation are depicted in Figure 5. This indicates potential deficits in cytoskeletal and synaptic function at different stages of iMN maturation, including the regulation of AS and gene expression.

Together, these findings suggest that ALS-associated risk factors may be developmentally programmed and contribute to motor neuron vulnerability well before overt degeneration occurs. Early cytoskeletal and synaptic dysfunction should be considered critical targets of ALS studies.

Previous studies have reported that a significant number of differentially spliced genes in ALS are RBPs (Ziff et al., 2023). Our analysis of motif enrichment around AS regions identified several RBPs as potential factors that bind to them (Figure 7B). Mutations in hnRNPs have previously been linked to neurodegenerative diseases, particularly ALS and frontotemporal dementia (FTD) (Bampton et al., 2020). All of the ALS datasets displayed differences in the expression of several previously identified RBPs (Figure 7C). One such RBP is PTBP1, which is enriched in regions flanking significant AS events and was dramatically downregulated in ALS-*FUS*, ALS-*TARDBP*, and ALS-*C9orf72* (Figure 7C). PTBP1 is an RBP and splicing regulator that is broadly expressed in non-neuronal and neuronal progenitor cells and represses neuronal-specific AS (Boutz et al., 2007; Makeyev et al., 2007; Ling et al., 2016). Although Ptbp1 was reported to play roles in neurogenesis and neuronal differentiation in mice (Zhou et al., 2020), it remains unknown whether PTBP1 causes motor neuron degeneration in ALS and requires further evaluation. A prior study showed that PTBP1 was identified as one of the FUS interactors, explaining its inability to inhibit pre-mRNA splicing in FUS-immunodepleted extract (Meissner et al., 2003). PTBP1 may contribute to neuronal death or perturb other mechanisms that maintain healthy neuronal function through interaction with other ALS-associated proteins.

In summary, our research identified changes in the transcriptome of ALS during motor neuron development and disease progression and created a comprehensive map of AS. These findings contribute to the growing body of evidence implicating dysregulated synaptic functions occurring before motor neuron degeneration in ALS and shared molecular characteristics across different ALS genes. These findings will also serve as a valuable resource for understanding the pathogenesis complexity, heterogeneity, and diversity of ALS.

## Methods

### Generation and culture of iPSCs

Full details of differentiation practices are provided in the supplemental information.

### Motor neuron differentiation

The motor neuron differentiation protocol was adapted from a published protocol (Du et al., 2015). Full details of differentiation practices are provided in the supplemental information.

### Electrophysiology

Whole-cell patch-clamp recording protocol was adapted from previously published work (Devlin et al., 2015). Full details of electrophysiology practices are provided in the supplemental information.

### qPCR

qPCR was performed on cDNA using qPCR SYBR Green Master Mix (UNIQ) with a QuantStudio 7 Flex Real-Time PCR System. Full details of qPCR practices are provided in the supplemental information.

### RNA-seq and differential gene expression analysis

Full details before RNA-seq are provided in the supplemental information. The reads were aligned to the GRCh37 reference genome with HISAT2 (2.2.1) after removing the adapter sequences and low-quality reads using Trimmomatic (0.39) (Bolger et al., 2014). Read counts were quantified using FeatureCounts in Rsubread (2.0.1) (Liao et al., 2014) using GENCODE GTF annotation version 19. Differential expression analysis of normalized gene expression was performed using DESeq2 (1.34.0) (Love et al., 2014), with a fold change ≥1.5 and false discovery rate (FDR) < 0.05 indicating significant DEGs. The transcriptome alteration pattern of the DEGs was clustered using the function cutreeDynamic from the R package dynamicTreeCut (1.63-1) (Langfelder et al., 2008). PROGENy signaling pathway activities were estimated using the decoupleR (2.0.1) (Badia-i-Mompel et al., 2022) and progeny (1.16.0) (Schubert et al., 2018) packages in R.

### AS analysis

RNA-seq data were used for differential splicing analysis using rMATS (4.1.2) (Shen et al., 2014). Significant AS events were filtered and categorized based on an IncLevelDifference (ΔPSI) absolute value greater than 0.1 and FDR < 0.05. Sashimi plots of AS events were generated using rmats2sashimiplot (2.0.3). Binding motif enrichment analysis was performed by rMAPS2 (2.0.0) (Hwang et al., 2020). Significantly spliced regions were used as the target regions for motif enrichment, whereas non-significantly spliced regions were used to estimate the background binding levels. Changes in the coding potential of alternatively spliced target genes in ALS subgroups were estimated using the R package MASER (1.12.1; https://github.com/DiogoVeiga/maser). All of the transcripts attached to alternatively spliced regions were annotated using GENCODE GTF annotation version 19.

### Enrichment analysis

GO analysis was performed using the clusterProfiler R package (4.2.1) (Wu et al., 2021). The overrepresentation of synaptic GO terms was estimated using the SynGO online portal (www.syngoportal.org) (Koopmans et al., 2019).

### Statistical analyses

All of the statistical tests were performed using GraphPad Prism 9 or R Studio. Data are presented as the mean ± SEM; in the case of *n* ≥ 3, Pearson’s correlation coefficient was employed for the correlation analyses. The Kruskal-Wallis test with Dunn’s correction for the ALS variables was performed to determine the distribution of exon inclusion levels. The function pairwise.wilcox.test() was used to calculate pairwise comparisons between group levels with corrections for multiple testing. Schematic illustrations were created using Biorender (https://biorender.com/). Data visualization of sequencing data analyses was performed in R using the VennDiagram (1.7.3), UpSetR (1.4.0), pheatmap (1.0.12), circlize (0.4.15), corrplot (0.92), lessR (4.3.0), scatterpie (0.2.1), and ggplot2 (3.4.2) packages.

## Resource availability

### Lead contact

Further information and requests should be directed to Dr. Wen-yuan Wang (wywang@sioc.ac.cn).

### Materials availability

The cell lines are available to all academic researchers worldwide upon signing a material transfer agreement. However, there are restrictions for industrial users, and specific fees may apply. No unique materials or reagents were generated or used in this study.

### Data and code availability

The research data are in principle shared and open according to the findable, accessible, interoperable, reusable principles. The RNA-seq data have been deposited into the Gene Expression Omnibus database with accession number GSE299997. This paper does not report original code.

## Acknowledgments

This work was supported by the National Key Research and Development Program of China (2024YFA1108000), the Shanghai Municipal Science and Technology Major Project (grant no. 2019SHZDZX02), the Shanghai Key Laboratory of Aging Studies (19DZ2260400 to W.-y.W.), and the 10.13039/501100001809National Natural Science Foundation of China (grant no. 82441053). We thank the staff members of the Integrated Laser Microscopy System (https://cstr.cn/31129.02.NFPS.CLMIS) at the National Facility for Protein Science in Shanghai (https://cstr.cn/31129.02.NFPS), for providing technical support and assistance in data collection and analysis.

## Author contributions

Conceptualization, C.-c.X., G.-m.M., and W.-y.W.; reprogramming of fibroblast, culture of iPSCs, and differentiation of iMNs, IF, and qPCR, C.-c.X.; cell culture, C.-c.X., J.L., F.L., and M.-f.G.; RNA-seq data analysis, G.-m.M. and B.-y.L.; electrophysiology, C.-c.X. and J.-y.W.; writing – original draft, G.-m.M. and C.-c.X.; writing – review and editing, G.-m.M., C.-c.X., W.-y.W., L.S., L.Z., Y.C., Y.-w.M., and G.-q.Y.

## Declaration of interests

The authors declare no competing interests.

## Supplemental information

Document S1. Figures S1–S7, Tables S1 and S2, and supplemental methods

Document S2. Article plus supplemental information
