# Supplementary material for: Integrated profiling of iPSC-derived motor neurons carrying C9orf72, FUS, TARDBP, or SOD1 mutations
Source: Stem Cell Reports. 2025 Oct 2;20(10):102649. doi: 10.1016/j.stemcr.2025.102649 (PMC12790725; doi:10.1016/j.stemcr.2025.102649)
Supplement: Document S1. Figures S1–S7, Tables S1 and S2, and supplemental methods [file mmc1.pdf]

Stem Cell Reports, Volume *20*

Supplemental Information

Integrated profiling of iPSC-derived motor neurons carrying *C9orf72*, *FUS*, *TARDBP*, or *SOD1* mutations

Guo-ming Ma, Cong-cong Xia, Bo-yu Lyu, Jie Liu, Fang Luo, Ming-feng Guan, Jun-ying Wang, Li Sun, Lin Zhang, Yan Chen, Ying-wei Mao, Guo-qiang Yu, and Wen-yuan Wang

Supplemental information

1. Supplemental figures and tables

1.1. Figures S1-S7

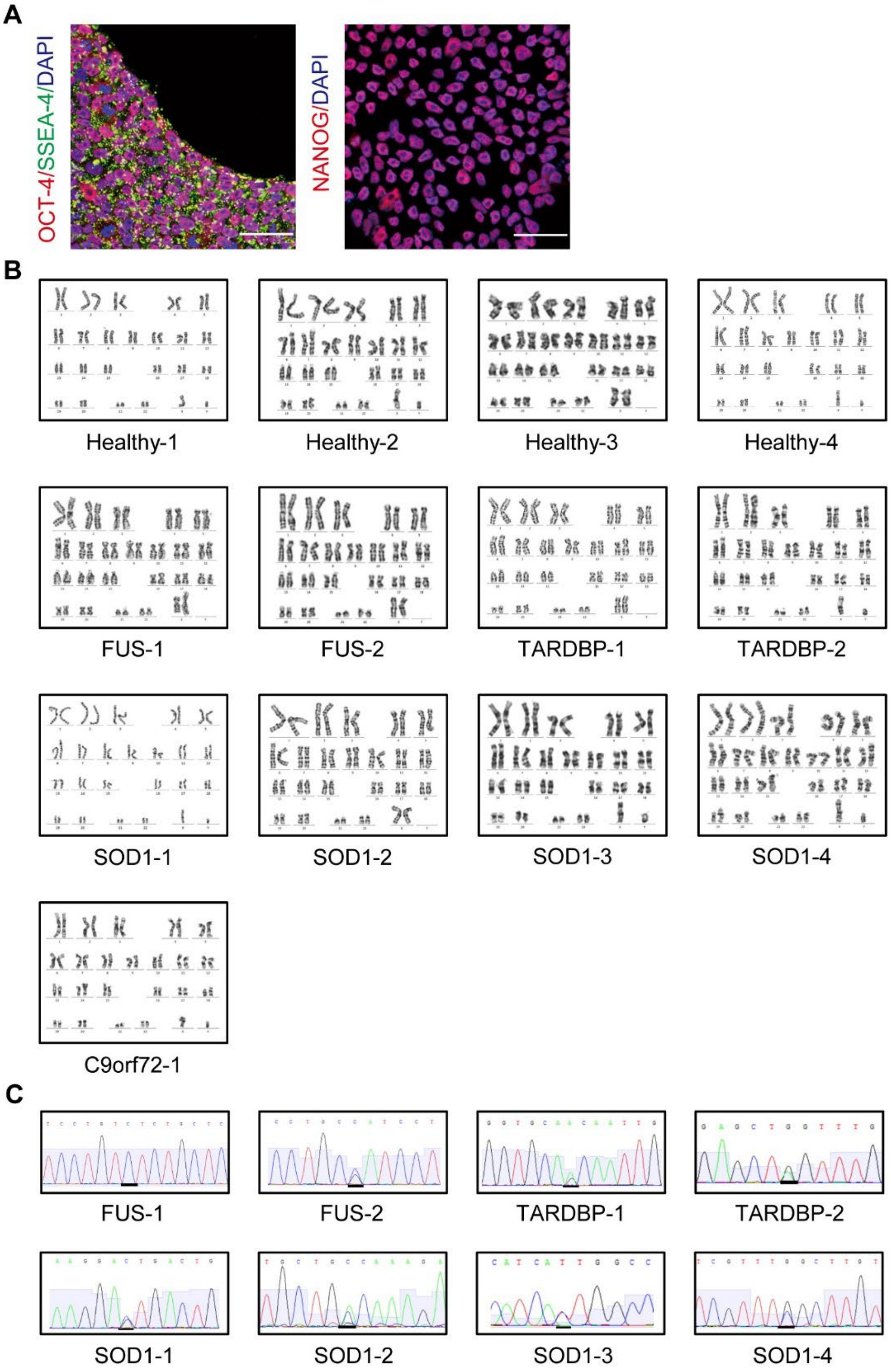

Figure S1. Characterization of iPSCs. (**A**) Immunofluorescence of pluripotent markers (OCT4, SSEA-1, and NANOG) for iPSCs. The scale bar represents 50 µm. (**B**) G-band karyotype analysis of iPSCs from controls and patients with ALS. (**C**) Confirmation of disease mutation of iPSCs from controls and patients with ALS.

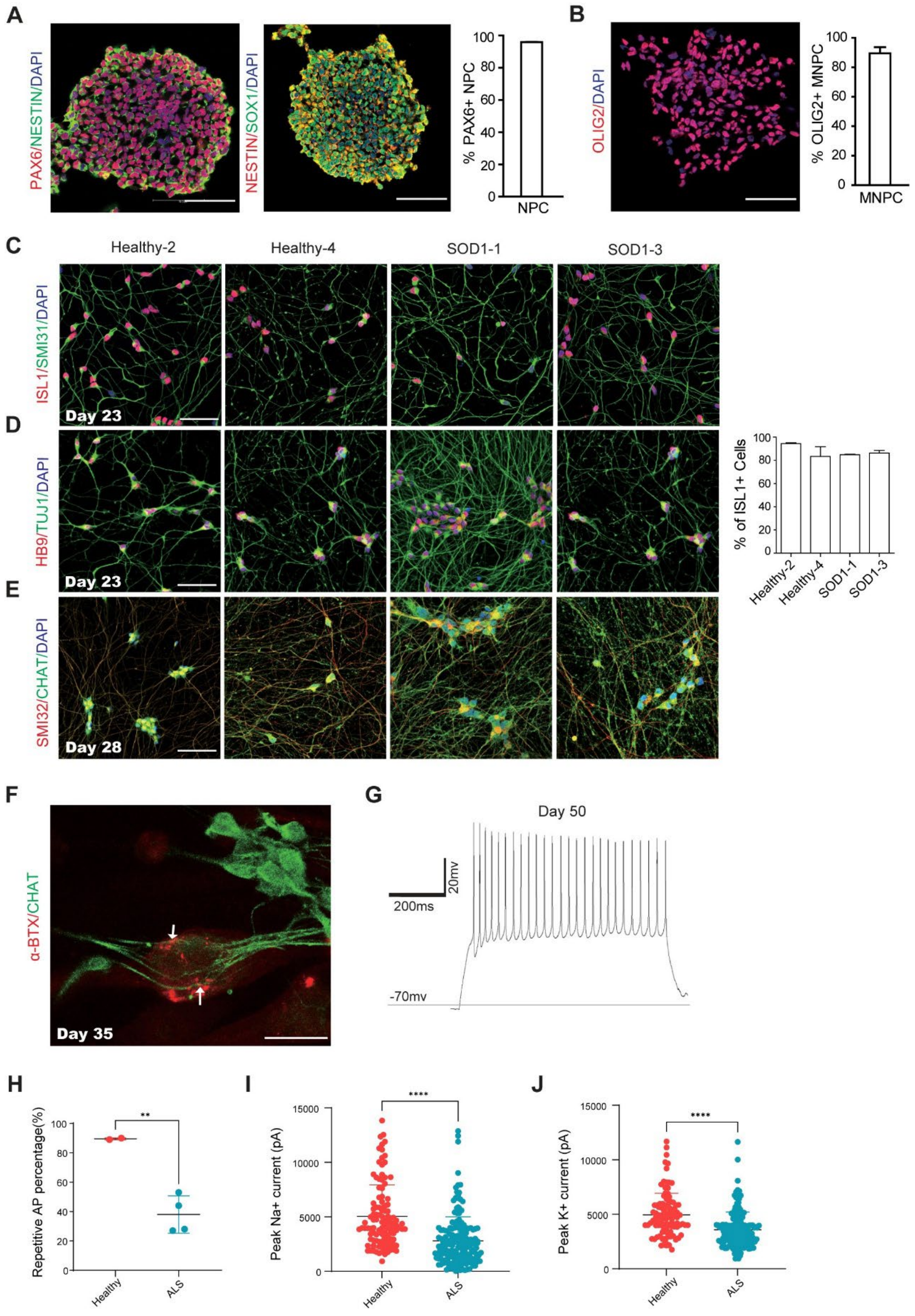

Figure S2. Characterization of iMN differentiation. (**A**) Representative images of SOX1+/PAX6+/NESTIN+ NPCs after 6 days of culture under the CHIR + SB + LDN condition. Cell nuclei were stained with DAPI (blue). Quantification of PAX6+ cells is shown. The scale bar represents 50 μm. (**B**) Representative images of pure OLIG2-

positive motor neuron progenitors (MNPs) on day 12. Cell nuclei were stained with DAPI. The scale bar represents 50 μm. (**C**–**E**) Representative images of the two motor neuron transcription factors ISL1/HB9, the functional motor neuron marker CHAT/SMI32 and the pan neuronal marker TUJ1. The scale bar represents 50 μm (left). The average percentage of ISL1+ motor neurons in healthy controls and ALS lines. No statistically significant difference was found (mean ± SEM; *n* = 3 independent experiments; Student's t-test; n.s., not significant) (right). (**F**) Representative images of iMNs, stained with the CHAT antibody (green), formed neuromuscular junctions labeled with α-bungarotoxin (α-BTX, red) when co-cultured with myotubes. The scale bar represents 20 μm. (**G**) An example voltage trace illustrates the response to current injections and highlights the generation of APs triggered by depolarizing current injections. (**H**) Proportion of cells in each AP firing category in iMNs from Healthy and ALS lines at weeks 8–9 post-plating. Each point represents an individual cell line: Healthy lines (n = 2), and ALS lines (n = 4). (**I**) Peak fast, inactivating Na^+ currents plotted from iMNs at weeks 8–9. (**J**) Peak K+ currents plotted from iMNs at weeks 8–9 (Student's t-test; $^{**}p < 0.01$; $^{***}p < 0.001$; $^{****}p < 0.0001$). For (**H**-**J**), healthy lines include healthy-1 (58 cells), and healthy-2 (61 cells); ALS lines include *FUS*-1 (52 cells), *SOD1*-1 (41 cells), *C9orf72*-1 (47 cells) and *TARDBP*-1 (41 cells). Cells from each cell line were derived from three independent batches of differentiation experiments. For (**H**), n represents the number of cell lines. For (**I**-**J**), n represents the number of individual cells recorded per line: Healthy (n = 119), and ALS (n = 181).

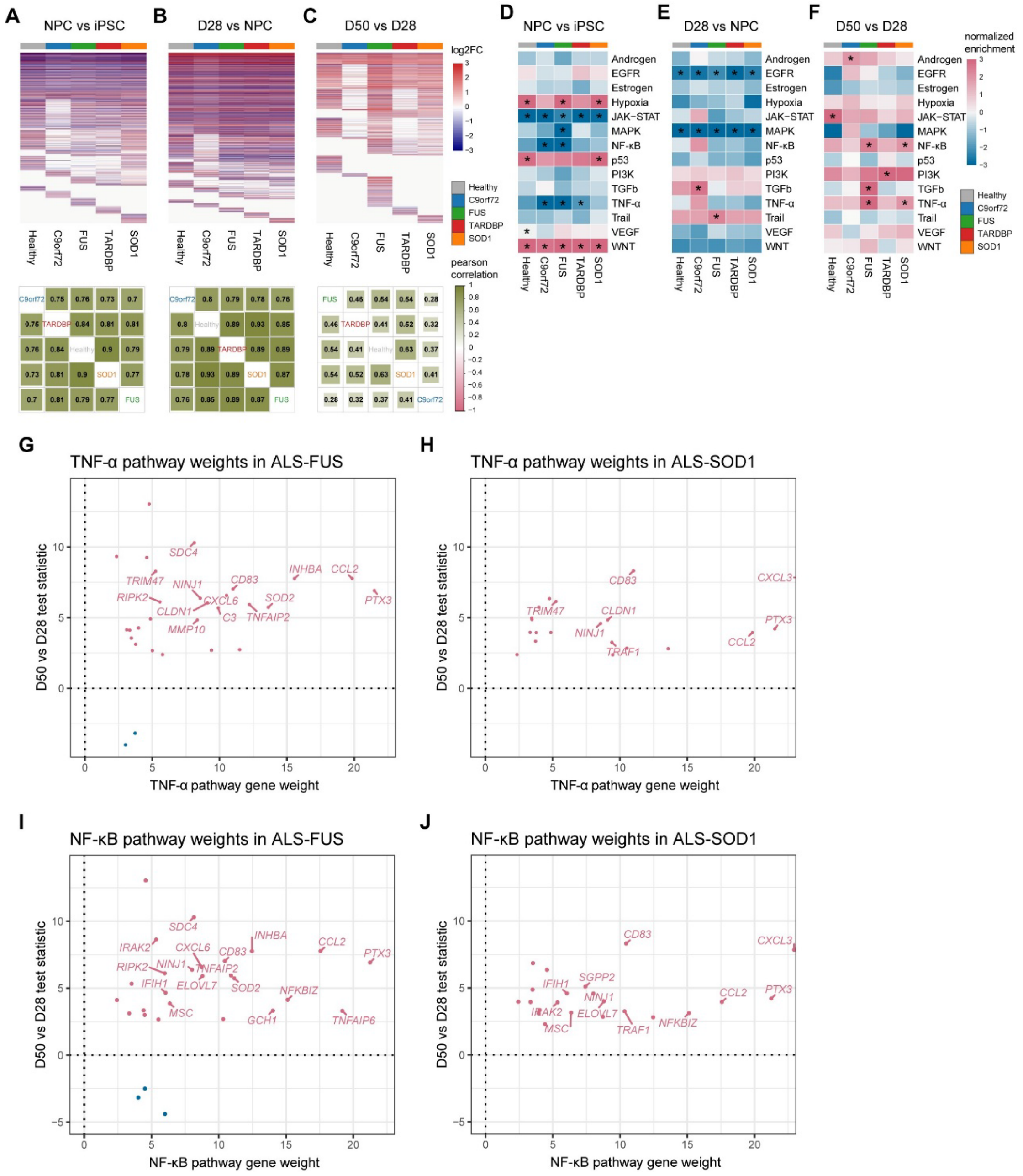

Figure S3. Temporal profiles of transcriptome alterations between each stage of iMN development and maturation. (**A–C**) Upper, heatmaps of differentially expressed genes in at least one genetic background between two consecutive developmental stages. Lower, the Pearson's correlation coefficient for transcriptome-wide changes between two genetic backgrounds in NPCs compared to iPSCs (**A**), D28-iMNs compared to NPCs (**B**), and D50-iMNs compared to D28-iMNs (**C**). (**D–F**) PROGENy signaling pathway activities in NPCs compared to iPSCs **(D)**, D28-iMNs compared to NPCs (**E**), and D50-iMNs compared to D28-iMNs (**F**) in healthy controls and ALS subgroups. Pathways that increase in later stages are red, while pathways that

decrease are blue. Statistics are from the weighted mean method (enrichment test; *$p < 0.05$). (**G**) Expression changes of TNF-α pathway genes in ALS-*FUS* (**G**) and ALS-*SOD1* (**H**), and NF-κB pathway genes in ALS-*FUS* (**I**) and ALS-*SOD1* (**J**) in D50-iMNs versus D28-iMNs according to their PROGENy weights. Genes in D50-iMNs that increase pathway activity are colored red, while those that decrease pathway activity are colored blue.

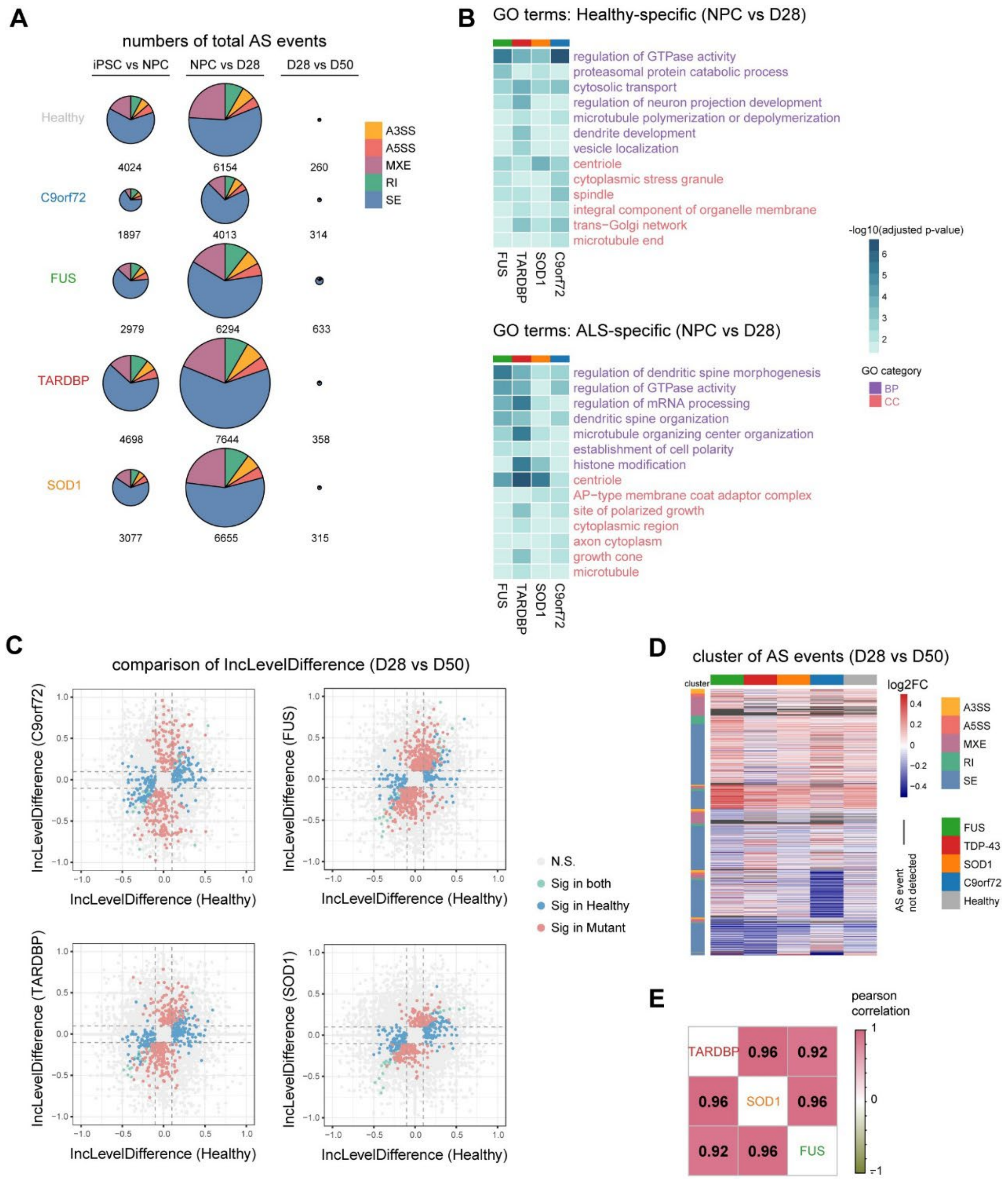

Figure S4. Aberrant splicing events during iMN development and maturation. (**A**) Pie charts representing counts of splicing events in each stage of iMN development compared to former stages. Chart areas are proportional to the total numbers of AS types at each stage. (**B**) Common GO terms associated with genes only differentially alternative spliced in healthy samples (left) from NPC to day 28 transition compared to corresponding ALS subgroups and vice versa (right). GO BP is colored purple, and GO cellular component (CC) is colored red. (**C**) Scatter plot of pair-wise comparisons of inclusion level differences in AS events detected in ALS-

C9orf72, ALS-*FUS*, ALS-*TARDBP*, and ALS-*SOD1* iMNs versus healthy controls. AS events show significant changes in both subgroups (green), the specific to healthy control subgroup (blue), and the specific to ALS subgroup (red). (**D**) Heatmap showing inclusion level differences in AS events significant in at least one iMN subgroup from included (red) to excluded (blue) (1484 events in 1055 genes). AS events that are not detected as significant are depicted by dark grey. (**E**) Correlation of inclusion level differences in AS events significant in at least one of ALS-*FUS*, ALS-*TARDBP*, or ALS-*SOD1* subgroups.

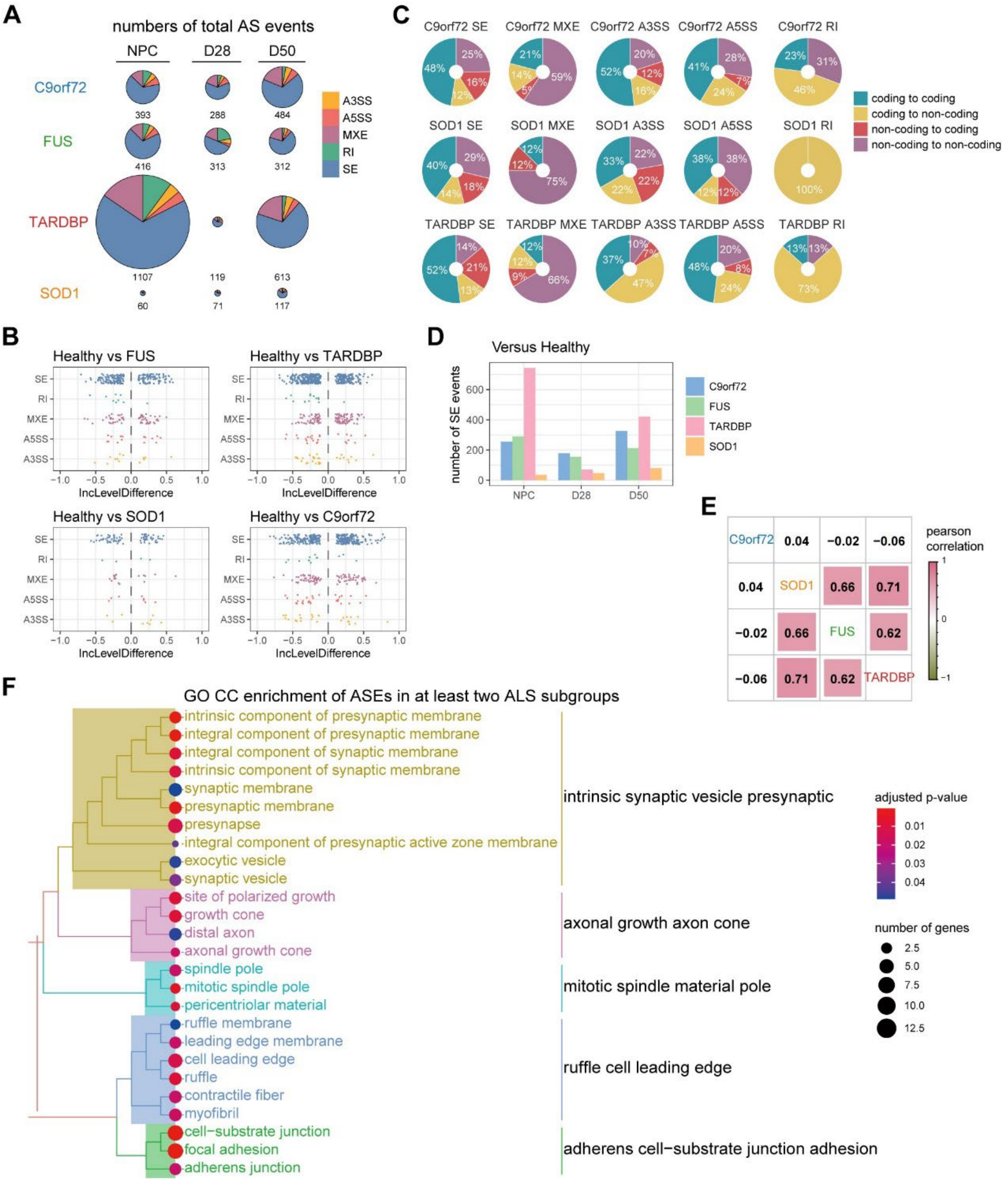

Figure S5. Temporal alternative splicing alterations in ALS iMNs. (**A**) Pie charts representing counts of splicing types in ALS subgroups at distinct stages of motor neurogenesis compared to healthy controls. Chart areas are proportional to the total numbers of events at each stage. (**B**) Jitter plot displaying distributions of included (IncLevelDifference > 0) and skipped (IncLevelDifference < 0) splicing events of all of the AS types in ALS iMNs compared to healthy controls. (**C**) Pie charts displaying the distribution of annotated transcripts with changes in their potential protein-coding ability, influenced by AS events in ALS-*TARDBP*, ALS-*SOD1*, and ALS-*C9orf72* iMNs

on day 50. Coding potentials were calculated by mapping splicing events to transcripts. (**D**) Bar graphs illustrating the counts of SE and MXE in ALS subgroups from NPC to D50-iMN. (**E**) Heatmap illustrating the correlation of inclusion level differences in AS events significant in at least one of the four ALS subgroups. Larger, darker squares denote a higher Pearson's correlation coefficient. (**F**) Tree plot depicting enriched GO CC terms of genes showing AS significant changes in at least two ALS subgroups.

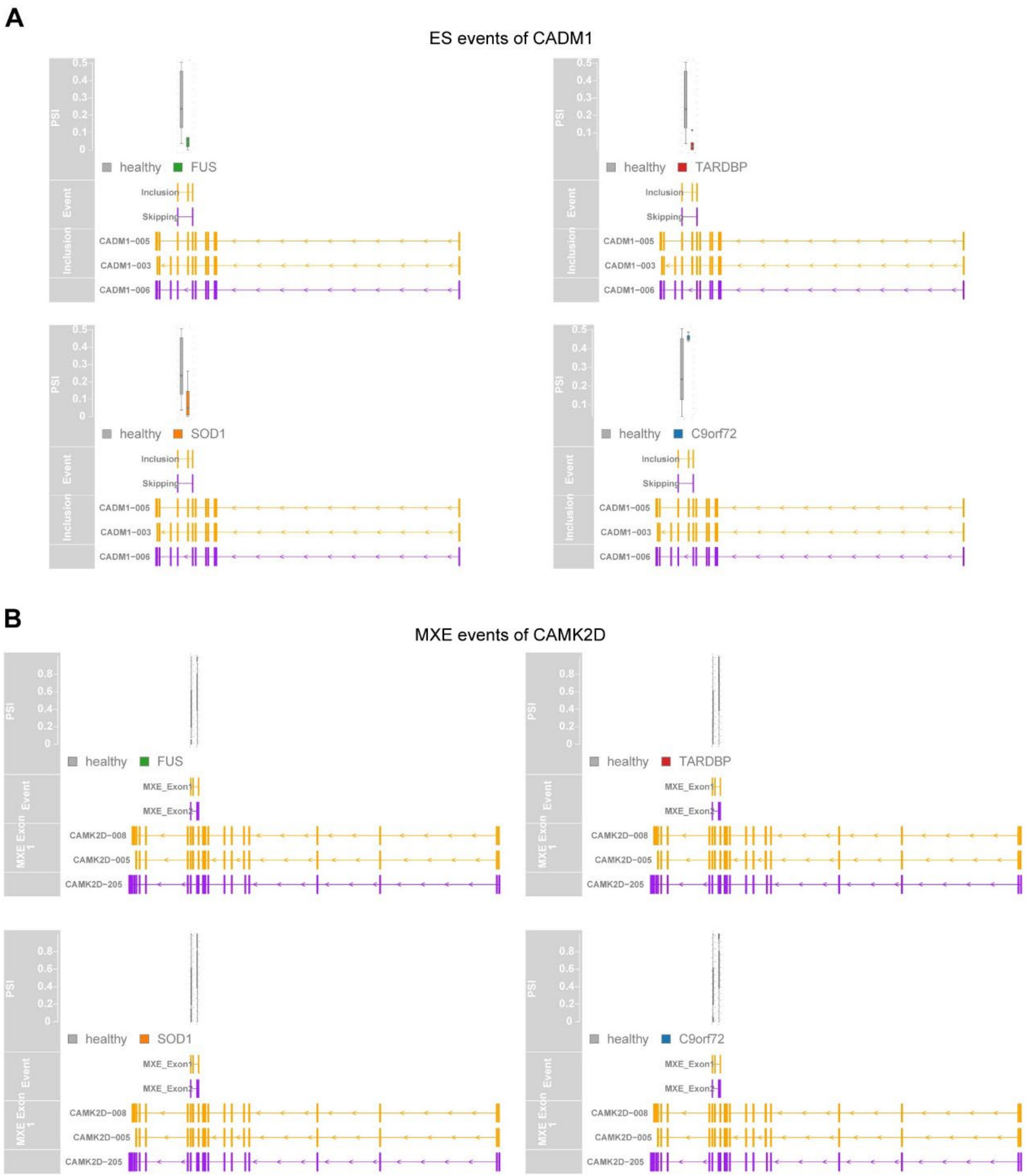

Figure S6. Aberrant AS events of *CADM1* and *CAMK2D* in ALS iMNs on day 50. (**A**) Exon skipping events of *CADM1* in ALS iMNs on day 50. (**B**) Mutually exclusive splicing events of *CAMK2D* in ALS iMNs on day 50. Boxplots show the difference in inclusion levels (PSI) between healthy and ALS subgroups. The event track showing the AS event with the flanking region, matches the annotated transcript tracks below.

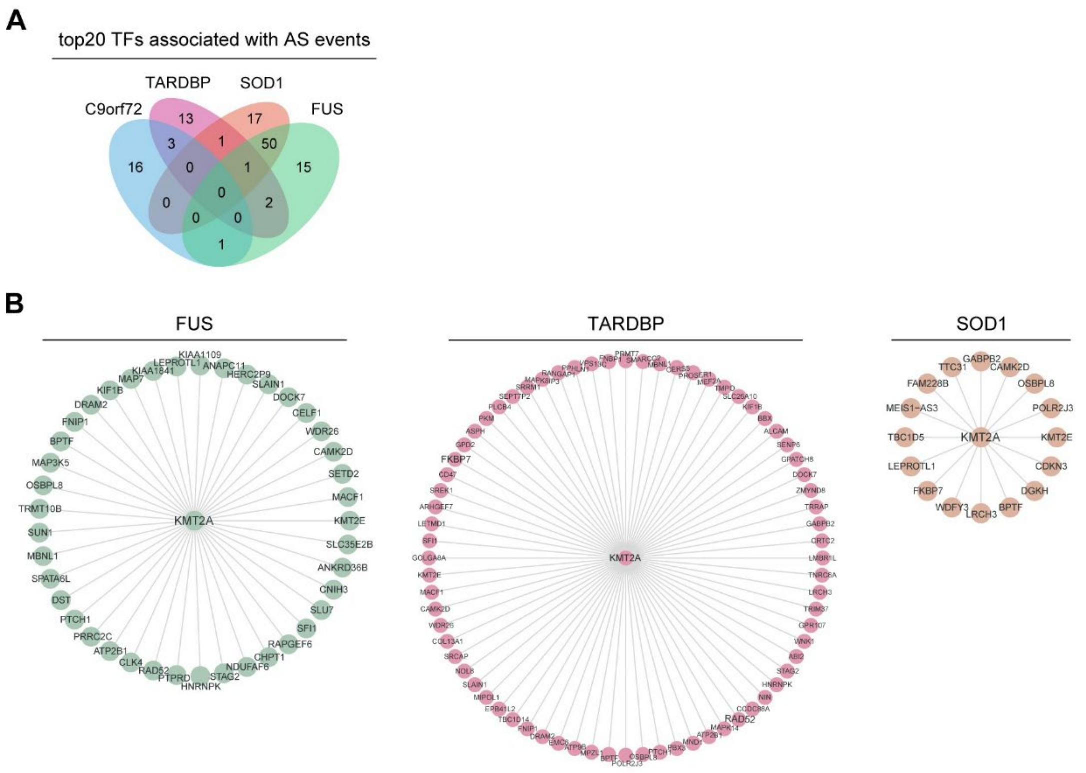

Figure S7. TF regulation of aberrant AS events in ALS iMNs on day 50. (**A**) Venn diagram displaying the number of transcription factors (TFs) associated with AS events that overlap among ALS iMNs. (**B**) Networks of key TF KMT2A and AS gene interactions. TF nodes are shown as circles in the center, and their target genes are shown as green (*FUS*), red (*TARDBP*), and orange (*SOD1*) dots. TF-to-AS gene edges are shown as gray lines between nodes.

1.2. Tables S1-S2

Table S1, related to Figure 1A: Details of fibroblasts from healthy donors and ALS patients used in this study.

Fibroblast cell line	Disease revelance	Genotype	Donor Age	Sex	Institution	Age of Onset	Site of Symptom Onset	Catalog ID
Healthy-1	Healthy	Healthy	40	Male	Coriell	-	-	ND07189
Healthy-2	Healthy	Healthy	40	Female	Coriell	-	-	AG09022
Healthy-3	Healthy	Healthy	34	Female	Coriell	-	-	AG07121
Healthy-4	Healthy	Healthy	New Born	Male	ATCC	-	-	CRL2522
SOD1-1	fALS	*SOD1*-L38V	47	Male	Coriell	44	Limb-lower	ND29523
SOD1-2	fALS	*SOD1*-D90A	50	Female	Coriell	44	Limb-upper	ND29149
SOD1-3	fALS	*SOD1*-I133T	55	Male	Coriell	49	Limb-upper	ND29422
SOD1-4	fALS	*SOD1*-L144P	51	Male	Coriell	48	Limb-upper	ND29415
FUS-1	fALS	*FUS*-H517Q	50	Female	Coriell	43	Limb-lower	ND39027
FUS-2	fALS	*FUS*-R521G	47	Female	Coriell	46	Limb-upper	ND40077
TARDBP-1	fALS	*TARDBP*-A382T	62	Female	Coriell	-	-	ND41003
TARDBP-2	fALS	*TARDBP*-G298S	64	Male	Coriell	62	Limb-lower	ND32947
C9orf72-1	fALS	*C9orf72*-6751J	52	Male	Coriell	50	Limb-lower	ND06751

Table S2, related to Figure 2F: PCR primers used in this study.

Gene	Forward	Reverse
ATP1A3	AAGGAGGTGGCTATGACAGAG	GTGAGTGCGTTAGGCCCAT
ATP1A2	GGGCACAGATATGGTCCCTG	TTGTCCGTCTGGGAGTTTCG
ACTIN	CCTCGCCTTTGCCGATCC	GAGTCCATCACGATGCCAGT
TFRC	GGACGCGCTAGTGTTCTTCT	CATCTACTTGCCGAGCCAGG

2. Supplemental Experimental Procedures

Generation and culture of iPSCs

Fibroblasts from patients with ALS carrying mutations and healthy controls were obtained from the Coriell Institute for Medical Research and ATCC (table S1). Details of the lines are presented in Table S1. These fibroblasts were reprogrammed into iPSCs using the non-integrating Sendai virus (A16517, Thermo fisher scientific). The reprogrammed iPSCs were maintained on Matrigel (354277, Corning) with Essential 8 Medium (A1517001, Thermo fisher scientific) and passaged using Gentle Cell Dissociation Reagent (# 100-0485, Stemcell). For routine passaging of hPSCs, cells are passaged by incubation (5 min, RT) with Gentle Cell Dissociation Reagent, followed by neutralization in Essential 8 Medium and replating at 1:6 ratio onto fresh Matrigel coated vessels, with daily Essential 8 medium exchanges to preserve characteristic undifferentiated colony morphology. For iPSCs freezing, cells are suspended in 10% DMSO+90% KnockOut™ Serum Replacement(10828028, Thermo fisher scientific), cooled gradually to -80℃, then stored in liquid nitrogen. For iPSCs thawing, vials are quickly warmed in a 37℃ water bath, diluted with culture medium, and centrifuged to remove cryoprotectants.The Master Cell Bank (MCB) for iPSCs is established at passage 15 (P15), representing an ideal balance between sufficient cell expansion and maintained genomic integrity. The Working Cell Bank (WCB) is subsequently generated at passage 20 (P20), extending only five passages beyond the MCB to ensure experimental consistency and minimize culture-induced variability. For routine experiments, iPSCs were maintained within the passage 20-25 (P20-P25) window, with all working cells strictly limited to below passage 25 (P25) to preserve genomic integrity and pluripotency. Sterility (by direct immersion in liquid culture) and mycoplasma testing (by PCR) were performed for each batch.

Motor neuron differentiation

The motor neuron differentiation protocol was adapted from a published protocol (Du et al., 2015). iPSCs between passages 20–25 were differentiated into neural progenitor

cells (NPCs) in chemically defined N2B27 medium, consisting of DMEM/F12 (11320033, Thermo fisher scientific), Neurobasal (21103049, Thermo fisher scientific) at 1:1, 0.5× N2 (17502048, Thermo fisher scientific), 0.5× B27 (A3582801, Thermo fisher scientific), 0.1 mM ascorbic acid (A4403, Sigma), 1× Glutamax (35050061, Thermo fisher scientific) and 1×penicillin/streptomycin (10378016, Thermo fisher scientific), β-mercaptoethanol (21985023, Thermo fisher scientific). Subsequently, 0.2 μM LDN-193189 (S2618, Selleck Chemicals), 2 μM SB431542 (S1067, Selleck Chemicals), and 3 μM CHIR99021 (S1263, Selleck Chemicals) were added to the medium for 6 days for NPC induction. On day 7, NPCs were cultured with 1 μM retinoic acid (RA, R2526, Sigma), 1 μM Smoothened Agonist (SAG, S7779, Selleck Chemicals), 1 μM CHIR99021, 0.2 μM LDN193189, and 2 μM SB431542 in N2B27 medium for an additional 6 days and differentiated into OLIG2+ motor neuron progenitors (MNPs). OLIG2+ MNPs were cultured in suspension in N2B27 medium with 1 μM RA and 1 μM SAG for 6 days and differentiated into ISL1+ MNs. The ISL1+ MNs were dissociated into single cells with 0.25% trypsin (25200072, Thermo fisher scientific) and then plated on poly-L-ornithine (10 mg/mL, P4957, Sigma)/laminine (5 mg/mL, 23017015, Thermo fisher scientific) coated plates. After 10 days of culture with 1 μM RA, 1 μM SAG, 0.1 μM Compound E (565790, Merck), 10 μg/mL BDNF (AF-450-02-50UG, perprotech), 10 μg/mL GDNF (AF-450-10-50UG, perprotech), and 10μg/mL CNTF (450-13-20UG, perprotech), MNs were differentiated into mature CHAT+ MNs. The MNs were cultured in N2B27 for 22 days without any additions and then collected for experiments.

Neuromuscular junction innervation

The neuromuscular junction detection protocol was adapted from a published protocol (Du et al., 2015). The glass coverslips were treated with trimethoxysilylpropyldiethylenetri-amine (DETA, Sigma), following the protocol (Guo et al., 2011). C2C12 cells were cultured on treated glass coverslips coated with Matrigel. C2C12 cells were cultured in DMEM containing 10% fetal bovine serum (FBS) and then induced to form myotubes by switching to DMEM containing 10% horse serum.

On day 18, MNs were digested into single cells and plated on the induced myotubes for 10 days, after which the neuromuscular junctions were visualized by performing immunofluorescence of CHAT (1:50, Millipore) and α-BTX-594 (1:200, Sigma) staining.

Immunofluorescence

Cells were fixed with 4% paraformaldehyde in 1×PBS for 10 min at room temperature (RT) and then blocked with 10% normal donkey serum (Life Technologies) with 0.2% TritonX-100 in PBS for 1 h. The cells were then incubated with primary antibodies in 1% BSA+ 0.1% TritonX-100 in PBS overnight at 4°C and incubated with secondary antibodies for 1 h at RT. The primary antibodies that follow were used: OCT-4 (1:400; Cell Signaling), -SSEA-4 (1:500; Cell Signaling), NANOG (1:250; Abcam), PAX6 (1:1000; Abcam), NESTIN (1:500; Abcam), OLIG2 (1:1,000; Millipore), ISLET1 (1:1,000; Abcam), SMI31 (1:1,000; Covance), mouse anti-SMI32 (1:1,000; Covance), HB9 (1:1,00; DSHB), TUJ1 (1:1,000; Abcam), CHAT (1:200; Millipore), and α-BTX-594 (1:200; Sigma).

Electrophysiology

Whole-cell patch-clamp recordings were used to detect the firing properties of iPSC-derived MNs. The recording protocol was adapted from previously published work (Devlin et al., 2015). The artificial cerebral spinal fluid used consisted of 119 mM NaCl, 5 mM KCl, 1.25 mM $NaH_2PO4.2H_2O$, 26 mM $NaHCO_3$, 2 mM $CaCl_2$, 1 mM $MgSO_4$, 5 mM glucose, and 95% O_2/5% CO_2. Recording pipettes were filled with K-gluconate-based current clamp internal solution containing 130 mM K-gluconate, 10 mM KCl, 10 mM HEPES, 0.2 mM EGTA, 0.5 mM Na_3-GTP, 4 mM Mg_2-ATP, 10 mM Na-phosphocreatine, pH 7.2, and 290 mOsm. Electrophysiological data were analyzed using Clampfit10 (Axon Instruments). Whole-cell patch-clamp recordings showed four firing patterns in response to current injections: no firing, single firing, adaptive firing, and repetitive firing (Devlin et al., 2015). The repetitive firing was characterized by a train of action potentials (APs) lasting for the duration of the square current injection (1s), whereas adaptive firing comprised multiple APs that ceased before the end of the

current stimuli. Cells were categorized as adaptive if they could not fire repetitively in response to a series of applied current steps (Devlin et al., 2015). When measuring Na^+ currents, we applied a range of voltage steps ranging from 70 to 20 mV in 2.5-mV increments. These voltage steps lasted for 10 ms each and were applied at a holding potential of 60 Mv. When measuring K^+ currents, we applied a range of voltage steps (−70 to 40 mV in 10 mV increments, with a 500 ms duration) from a holding potential of –60 mV. Differences between healthy controls and ALS subgroups were analyzed using Student's t-test. P-values < 0.05 were considered to be significant. Data for Na^+ and K^+ currents are presented as the mean ± standard error of the mean (SEM).

RNA extraction and qPCR

TRIzol (Invitrogen) was used to extract total RNA. DNA digestion and reverse transcription were performed using the Hifair™ **III** 1st Strand cDNA Synthesis SuperMix for qPCR (YEASEN) according to the manufacturer's instructions. qPCR was performed on cDNA using qPCR SYBR Green Master Mix (UNIQ) with a QuantStudio 7 Flex Real-Time PCR System according to the manufacturer's instructions. The relative expression levels of target genes were measured using the ddCt method normalizing to control gene expression. ACTIN was used as a reference in the ALS-*SOD1*, ALS-*FUS*, and ALS-*TARDBP* subgroups. TFRC was used as a reference in the ALS-*C9orf72*. The primers used for qPCR are listed in Table S2.

RNA sequencing and differential gene expression analysis

Total RNA was extracted using TRIzol (Invitrogen). RNA purification, reverse transcription, and library construction were performed in Mingma Technologies Co., Ltd. (Shanghai, China) according to the manufacturer's instructions. RNA integrity were measured using an Agilent 2100 Bioanalyzer (Agilent). The mRNA-focused sequencing libraries from total RNA were prepared using the VAHTS mRNA-seq v3 Library Prep Kit (VAHTS, NR611). PolyA mRNA was purified from total RNA and then fragmented. The final cDNA libraries were sequenced on an Illumina HiSeq PE150 sequencing system following Illumina protocols provided by Mingma Technologies Co., Ltd. in Shanghai.

Gene-transcription factor (TF) interaction analysis

The ChEA3 web browser (Keenan et al., 2019) was used to infer upstream regulators of genes with differentially AS. The mean rank was used in this study.

3. Supplemental references

Devlin, A.-C., Burr, K., Borooah, S., Foster, J.D., Cleary, E.M., Geti, I., Vallier, L., Shaw, C.E., Chandran, S., and Miles, G.B. (2015). Human iPSC-derived motoneurons harbouring TARDBP or C9ORF72 ALS mutations are dysfunctional despite maintaining viability. Nat Commun *6*, 5999. https://doi.org/10.1038/ncomms6999.

Du, Z.-W., Chen, H., Liu, H., Lu, J., Qian, K., Huang, C.-L., Zhong, X., Fan, F., and Zhang, S.-C. (2015). Generation and expansion of highly pure motor neuron progenitors from human pluripotent stem cells. Nat Commun *6*, 6626. https://doi.org/10.1038/ncomms7626.

Guo, X., Gonzalez, M., Stancescu, M., Vandenburgh, H.H., and Hickman, J.J. (2011). Neuromuscular junction formation between human stem cell-derived motoneurons and human skeletal muscle in a defined system. Biomaterials *32*, 9602–9611. https://doi.org/10.1016/j.biomaterials.2011.09.014.
